# Memory modulation: Dominance of negative visual context over neutral verbal memory

**DOI:** 10.1371/journal.pone.0312042

**Published:** 2024-10-14

**Authors:** Stas Kozak, Noa Herz, Maya Tocker, Yair Bar-Haim, Nitzan Censor

**Affiliations:** 1 School of Psychological Sciences, Tel Aviv University, Tel Aviv, Israel; 2 Department of Neurology, Thomas Jefferson University, Philadelphia, Pennsylvania, United States of America; 3 Department of Psychology, Haifa University, Haifa, Israel; 4 Sagol School of Neuroscience, Tel Aviv University, Tel Aviv, Israel; Gabriele d’Annunzio University of Chieti and Pescara: Universita degli Studi Gabriele d’Annunzio Chieti Pescara, ITALY

## Abstract

Neutral memories can be modulated via intentional memory control paradigms such as directed forgetting. In addition, previous studies have shown that neutral visual memories can be modulated indirectly, via remember and forget instructions towards competing verbal memories. Here we show that direct modulation of neutral verbal memory strength is impaired by negative visual context, and that negative visual context is resistant to indirect memory modulation. Participants were directly instructed to intentionally remember or forget newly encoded neutral verbal information. Importantly, this verbal information was interleaved with embedded negative visual context. Results showed that negative visual context eliminated the well-documented effect of direct instructions to intentionally remember verbal content. Furthermore, negative visual memory was highly persistent, overcoming its sensitivity to indirect modulation shown in previous studies. Finally, these memory effects persisted to the following day. These results demonstrate the dominance of negative visual context over neutral content, highlighting the challenges associated with memory modulation in psychopathologies involving maladaptive processing of negative visual memories.

## Introduction

In daily life, we often experience events we wish to forget. Humans can actively forget unwanted memories through intentional memory control, such as directed forgetting (DF), in which individuals are instructed to remember or forget newly encoded information [1, 2]. Studies have shown that the instruction to forget, compared to the instruction to remember, prompts prefrontal inhibitory control over mnemonic hippocampal activity, resulting in decreased memory strength [1, 2]. Thus far, DF paradigms demonstrated reliable direct memory modulation effects for neutral stimuli [3–6]. Additional studies have shown that neutral memories can be modified indirectly during online processing [7–10], and post-encoding via mechanisms of memory competition [11].

Nevertheless, modulation of negative memories is a more challenging endeavor. Negative emotional memories show high resistance to deliberate suppression [12–14], and require extensive neuro-cognitive engagement [15–19]. Following their consolidation, adverse memories become even less susceptible to direct modulation [14, 20, 21]. Consolidated emotional memories become distributed across multiple neural networks [21], possibly persisting through involuntary recurrent reactivation, and in some cases result in pathological outcomes such as intrusive memories [22, 23]. In the same vein, effective regulation of negative memories can support resilience and mental health [24–26], and is key in the treatment of psychopathologies such as depression, fear disorders, and post-traumatic stress disorder (PTSD) [27–29].

**Fig 1:**
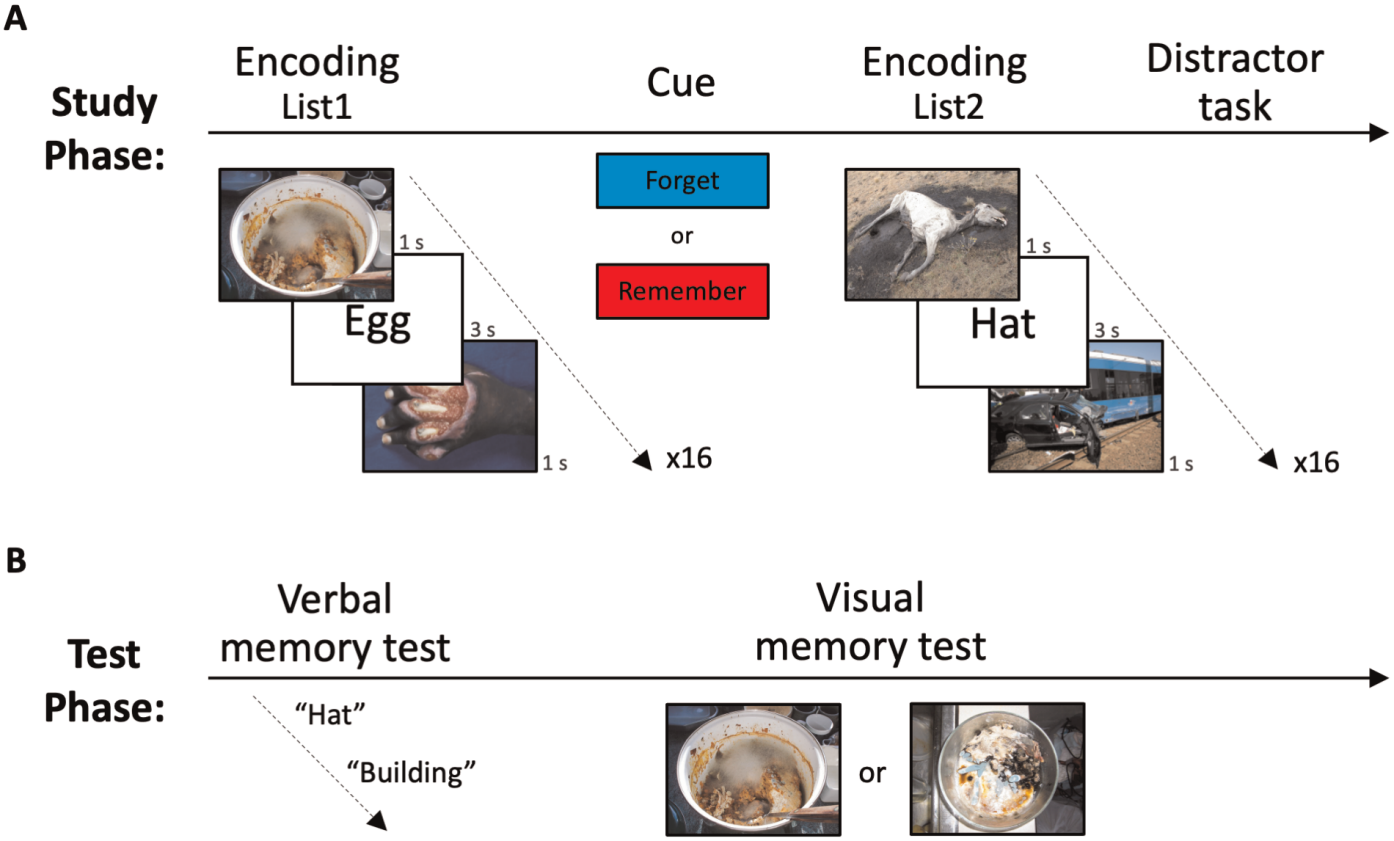
Study design. (A) Participants were instructed to study two lists of neutral words embedded within negative pictures. After studying List1, participants were presented with a cue to either forget or remember the studied words. (B) On the day of encoding and on the following day, we measured participant’s memory strength for the words (direct memory modulation), and the pictures (indirect memory modulation). The pictures illustrated are from the DIsgust-RelaTed-Images (DIRTI), and the Nencki Affective Picture System (NAPS) databases [35, 37].

Previously, we found that neutral visual context is susceptible to indirect memory modulation [11]. Here, we show that negative visual context attenuates direct modulation of neutral verbal memory strength, and that this negative visual context is resistant to indirect memory modulation. Participants studied two lists of neutral words, embedded within negative pictorial context. To test direct modulation of neutral memory, participants received a cue to either forget or remember the words from the list they had just studied. To evaluate indirect modulation of the emotional memory, participants did not receive any instructions regarding the negative pictures (Fig 1A) (see Materials and methods). After completing the study phase, we measured verbal and visual memory through free recall and two-alternative forced choice recognition tests, respectively (Fig 1B). To monitor memory persistence effects, participants returned to the lab on the following day to perform another verbal and visual memory test.

## Materials and methods

### Participants

Forty-three healthy volunteers (22 females, *M*_*age*_ = 23.84 years, *SD* = 2.45, *Range* = 18–30) were recruited through online and printed advertisements. All participants reported at least 6 hours of sleep the night before the experimental sessions, and reported no history of neurological, physical, or mental disorders. One participant was excluded due to difficulty in understanding task instructions and two participants did not attend the second day of the study, one due to the emotional content of the experiment and the other for medical reasons. Sample size was determined based on an averaged reported effect size (η^2^_p_ = 0.127, power = 0.95) found in previous list-method directed forgetting studies [30–32] and in accordance with previous experiments involving a similar paradigm (n = 40, see main experiment in [11]). Study recruitment was conducted between 2/6/2021-4/09/2022. Participants provided written informed consent prior to testing and were compensated $20 or course credit. The Tel Aviv University Institutional Review Board approved the study, and all methods were performed in accordance with the relevant guidelines and regulations.

### Procedure

Participants performed a directed forgetting paradigm, with emotionally negative pictures interleaved between the to be remembered/forgotten words [11]. Participants observed two lists of neutral words (16 per list), embedded within negative pictorial context (32 per list) [33–37], with each trial presenting a triplet of picture-word-picture (Fig 1A). To avoid semantic overlap between presented words and pictures, the studied words were of objects with minimum relation to the pictures that depicted negative scenes. Each word was presented for 3 sec and each picture for 1 sec. Inter-stimulus-interval (ISI) between trials was 3 sec. Both words and pictures were randomized across and within lists [11].

Participants were told that they are about to see a list of words interleaved with pictures, and were instructed to study the words for a future memory test. Importantly, these instructions were given only regarding the words (direct memory modulation), and not the pictures (indirect memory modulation). After studying the words of List1, participants received a cue to either forget (n = 20) or remember (n = 22) this list of words. All participants were instructed to remember the words of List2. To reduce recency effects and deliberate words memorization, participants solved arithmetic problems for 1 minute as a distractor before the test phase [38, 39] (Fig 1A).

Participants’ verbal and visual memory strength was measured immediately following the distractor task (Fig 1B). First, participants were asked to write as many words as they can recall from both studied lists (List1 and List2), including from the list they were instructed to forget (List1). There was no time limit for this test. Next, participants performed a visual memory test on all the pictures they have seen during the study phase. In each trial participants saw two pictures on the screen, one which previously appeared in the study phase, and a foil picture with the same semantic content and similar visual features. Participants had to choose the picture they have seen before and were asked to answer as quickly and as accurately as possible.

To monitor memory persistence effects, participants returned to the lab on the following day to perform another verbal and visual memory test on the same stimuli encoded the previous day. Participants performed the second day memory tests in the same laboratory room, with the same research assistant and approximately at the same time of the day. Following the memory test participants rated the valence of the study pictures [40] to assure that the pictures were perceived as negative. Participants were exposed to all studied negative pictures (N = 64) together with new 32 neutral valence pictures, operationalized as pictures with scores between 4 and 6 on a 1–9 Self-Assessment Manikin scale [11]. Participants’ ratings confirmed that the pictures were experienced as negative. Participants rated the study pictures as negative (*M* = 3.16, *SE* = 0.09) (rating lower than 4 on the Manikin scale), relative to neutral pictures, in both experimental conditions (*F*(1,38) = 378.389, *p*<0.001, η^2^_p_ = 0.909).

### Data analysis

Only exact recall of the studied words, or words with phonetically equivalent spelling mistakes (< 1.1%) were scored by the coder as correct answers in the verbal memory test. For each list of words and pictures we calculated the percent of correct responses. We then winsorized data points falling above two standard-deviations from the mean in each experimental condition and list to the highest score inside the range of -2<Z<2 [41]. In the picture recognition test, we excluded trials with a response time lower than 300 ms (< 0.001%) [11]. We also calculated, for each list, the average valence rating of the negative contextual pictures.

A two-way mixed ANOVA was performed for word or picture memory strength, with instruction (remember/forget) as a between-subjects factor and list (List 1, List 2) as a within-subject factor. We applied nonparametric permutation analysis for comparisons that did not withstand the equal variance assumption. Null results were additionally confirmed with a complimentary Bayesian analysis with instruction (remember/forget) as a between-subjects factor and list (List 1, List 2) as a within-subject factor. Bayesian analysis was performed using the statistical program JASP and its default priors (https://jasp-stats.org/).

## Results

### Memory performance day 1

#### Verbal memory

**Fig 2:**
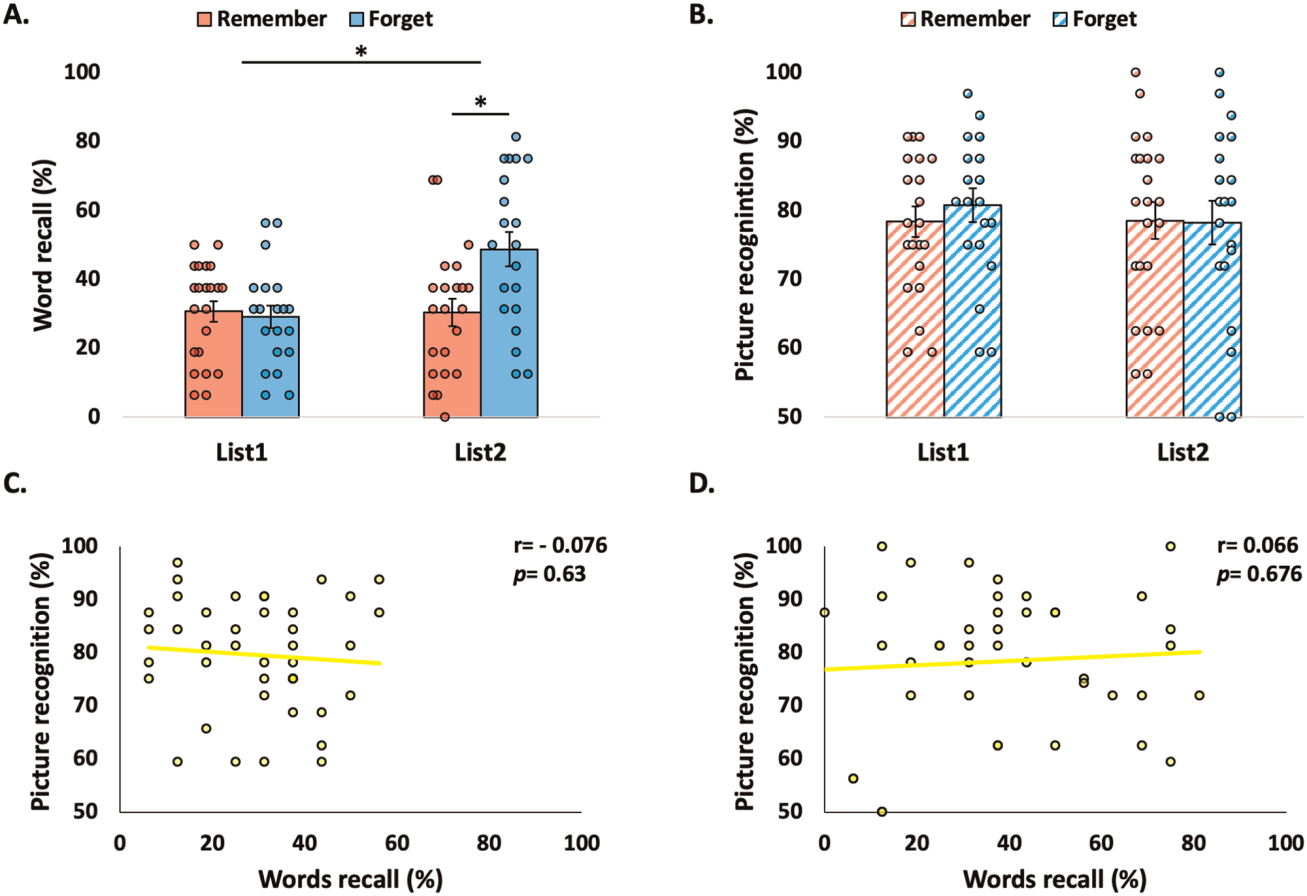
The dominance of negative visual context over neutral verbal memory. (A) Under negative context, the instruction to remember List1 words (light red) did not enhance verbal memory, compared to the instruction to forget the words (light blue). List2 verbal memory strength was greater following the instruction to forget the words (light blue), relative to the instruction to remember the words (light red). (B) Negative visual memory showed resistance to indirect modulation, with comparable visual memory strength in List1 versus List2 when participants were instructed to remember or to forget the words. (C) There was no correlation between verbal and visual memory strength for List1. (D) Similarly, there was no correlation between verbal and visual memory strength for List2. Error bars represent ±1 Standard error of the mean (S.E.M.) *p<0.05.

Verbal memory strength, under the influence of negative visual context, showed an instruction x list interaction (*F*(1,40) = 7.513, *p* = 0.009, η^2^_p_ = 0.158). Negative context eliminated the well-documented directed forgetting cost effect [42, 43] (Fig 2A, left panel), i.e., the beneficial effect of the instruction to remember the words compared to the instruction to forget the words, resulting in similar List1 verbal memory strength under both conditions (*t*(40) = -0.366, *p* = 0.717, d = -0.113; further supported by a complimentary Bayesian analysis, BF_01_ = 3.125, error % = 0.014). This was contrary to neutral visual context, which did not affect DF memory modulation in our previous study [11]. However, under negative context, verbal memory strength on List2 was greater following the instruction to forget the words, compared to the instruction to remember the words (*t*(40) = 2.89, *p* = 0.006, d = 0.896), also known as the directed forgetting benefit effect [44, 45] (Fig 2A, right panel).

#### Negative visual memory

Next, we asked whether instructions to remember/forget the words indirectly modulated negative visual memory strength. Results showed that negative visual memory was resistant to the established indirect modulation effect shown with neutral memories [11], with comparable visual memory strength in List1 relative to List2 when instructions were to remember or forget the words (*F*(1,40) = 0.486, *p* = 0.49, η^2^_p_ = 0.012; further supported by a complimentary Bayesian analysis, BF_01_ = 29.861, error % = 1.691) (Fig 2B). Additionally, in contrast to a previous study with neutral visual context [11], when using negative visual context there was no significant correlation between verbal and visual memory strength neither for List1 (*r* = -0.076, 95% CI[0.225,-0.36], *p* = 0.63, BF_01_ = 4.651) (Fig 2C) nor for List2 (*r* = 0.066, 95% CI[0.351,-0.234], *p* = 0.676, BF_01_ = 4.78) (Fig 2D).

### Memory performance day 2

#### Verbal memory

**Fig 3:**
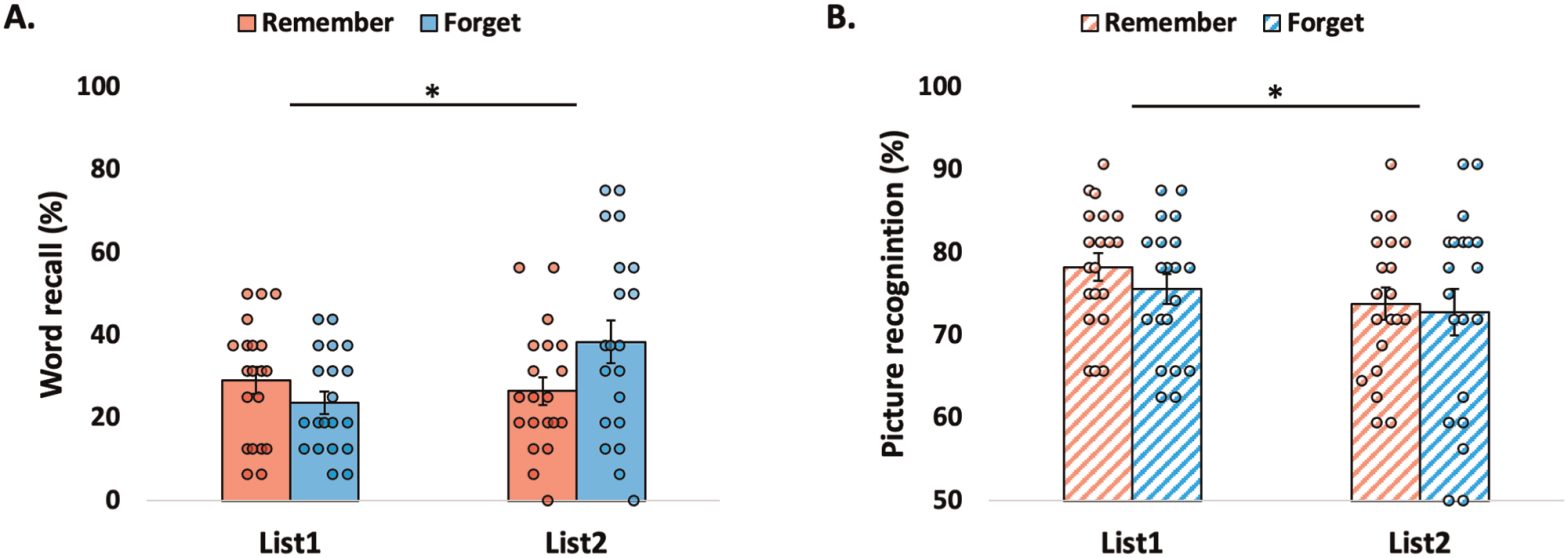
The dominance of negative visual context over neutral verbal memory persisted to the following day. (A) Verbal memory strength remained similar for words under the remember and the forget conditions for the List1 and enhanced after the instruction to forget the words for List2. (B) Resistance of negative visual memory to indirect modulation also persisted to the next day. There was a general primacy effect for negative contextual pictures, with pictures from List1 remembered better than pictures from List2 under both types of instructions. Error bars represent ±1 Standard error of the mean (S.E.M.) *p<0.05.

These results remained stable in the subsequent day of testing, with verbal memory strength showing an instruction x list interaction (*F*(1,38) = 4.96, *p* = 0.032, η^2^_p_ = 0.115). Verbal memory strength remained similar between the remember and forget conditions for List1 (*t*(38) = -1.257, *p* = 0.216, d = -0.397; further supported by a complimentary Bayesian analysis, BF_01_ = 1.736, error % = 0.007), again indicating an elimination of the directed forgetting cost effect (Fig 3A). Additionally, verbal memory was marginally better following the forget instruction compared to the remember instruction for List2 (*t*(38) = 1.914, *p* = 0.064, d = 0.61) (Fig 3A).

#### Negative visual memory

The resistance of negative visual memory to indirect modulation also persisted to the next day (*F*(1,38) = 0.319, *p* = 0.575, η^2^_p_ = 0.008; further supported by a complimentary Bayesian analysis, BF_01_ = 2.412, error % = 1.564). In addition, there was a general primacy effect for negative visual memory, with better visual memory strength for List1 relative to List2 under both types of instructions (*F*(1,38) = 6.135, *p* = 0.018, η^2^_p_ = 0.139) (Fig 3B).

## Discussion

The results emphasize the dominance of negative visual context memory over neutral content memory. Under negative context, the instruction to remember the words did not improve verbal memory relative to the instruction to forget the words. Additionally, negative visual memory was not affected by indirect modulation, with comparable visual memory strength under either instruction. Taken together, the current results suggest that negative context impairs memory of embedded neutral details and is resistant to indirect memory modulation.

The suggestion that negative visual context impairs related neutral memory is in line with previous findings. Negative events usually attract more attention than neutral events, leading to increased neural activity associated with the perception and encoding of the negative event, resulting in better memory [46, 47]. Even when negative and neutral information is attended similarly, the encoding of negative arousing memories is often prioritized at the expense of related neutral memory encoding [48–50]. Interestingly, negative context can also strengthen related neutral memories [51–53], for example when neutral information is linked to a threating context [54, 55]. Of note, while negative context reduced verbal memory strength for the remember condition, it did not affect verbal memory strength for the forget condition. These results are consistent with similar findings from the DF item-method paradigm [56, 57], suggesting that negative context disrupts memory upregulation (remember condition) and not downregulation (forget condition). While we did not find a DF cost effect for memory recall, this effect is mostly preserved for memory recognition [56, 57], indicating that negative context might impair memory retrieval more than memory encoding.

Negative visual context could also strengthen verbal memory following instructions to forget the words. Several underlying brain mechanisms may account for these effects, and should be further examined in future studies. Enhanced memory after intentional forgetting relates to a decrease in frontoparietal EEG alpha power [58, 59], possibly reflecting a reset in memory encoding [45]. Negative visual stimuli similarly induce a decrease in alpha power [60–63]. Additionally, negative stimuli can promote amygdala driven hippocampal inhibition [64–66], similar to hippocampal downregulation elicited by the forget instruction [1, 2]. The results of the current study suggest that intentional forgetting together with negative context synergistically promotes subsequent memory, while such an effect is not evident under neutral context [11].

The current results demonstrate the dominance of negative visual memory over neutral verbal memory, with neutral verbal memory strength impaired by negative visual context and negative visual context resistant to indirect memory modulation. In addition, this dominance may have abolished the correlation between negative visual memory strength and neutral verbal memory strength. Negative visual memories engage sensory and medial temporal lobe areas to support better encoding and memory vividness, often on the expense of neutral memory processing [23, 67]. Negative memories dominate through arousal and valence driven processes [68–71]. High arousal triggers automatic noradrenergic response in the amygdala to alter hippocampal activity in favor of the arousing memory, while negative valence activates a controlled fronto-hippocampal process to prioritize negative memory. These visual memories are forgotten more slowly compared to neutral visual memories, through support of amygdala driven item–emotion bindings and repeated negative memory reactivation [23, 72]. Here, we demonstrate a post-consolidation advantage for negative visual memory encoded in the first part of the learning session. In psychopathology distressing visual memories are especially persistent, and recurrent involuntary retrieval of aversive imagery can last many years after experiencing a traumatic event [65, 73].

Nevertheless, it is important to note the limitations of the current study. We examined the dominance of pictures with negative valence; however, it is possible that arousal induced by the negative pictures affected the current results. While high arousal is believed to prioritize memory encoding of the arousing event over related content [47], negative valence is thought to enhance memory for the aversive event [74]. Future studies should disentangle the contribution of valence and arousal on negative visual memory dominance. Additionally, we found that negative visual memories were resilient to indirect memory modulation on recognition memory tests, nevertheless it is possible that our method indirectly affected negative visual memory recall [75, 76]. Finally, this study included only words and pictures, portraying a simplified model of everyday life events. Future studies should explore the dominance of negative visual memories in more ecological settings such as augmented and virtual reality [77, 78].

## Conclusions

Our results exhibit the superiority of negative visual context memory over neutral content memory, and negative visual memory durability to indirect memory modulation. These findings further emphasize the current challenges to effectively downregulate and modulate pathological distressing visual memories such as intrusive memories [65, 73]. Future research could test alternative memory modulation methods to overcome the noted dominance and resistance of negative visual memories. For example, positive verbal memories can attenuate negative emotional responses [79, 80], and possibly alter negative visual memory strength. Additionally, non-invasive brain stimulation can modulate human learning and memory [81–83]. Recent studies have shown that such neuromodulation using repetitive transcranial magnetic stimulation (rTMS) can reduce the intensity of reactivated intrusive memories and fear memories in non-clinical populations [84, 85]. This study was conducted in healthy participants rather than clinical populations. It remains to be determined whether similar effects would occur for example in patients with PTSD. Such patients are likely to experience greater long term memory impairment under negative context, compared to healthy participants, as they perform poorer on working memory tasks with negative emotional context [86–88], and have difficulties in suppressing unwanted memories [89, 90]. Elevating the capability of PTSD patients to withstand emotional distress and intentionally control memories, by targeting reward and prefrontal networks, could be an important step in overcoming the dominance of negative memories in psychopathology [25, 91–93]. Taken together, we suggest that interventions in patients, geared to modulate negative visual memories, would require multiple sessions spanning a longer duration. Therefore, the development of strategies combining behavioral and neuromodulation approaches described here may open new avenues for alleviating the burden of maladaptive negative memories in clinical populations.
